# Depression, anxiety, and stress among inflammatory bowel disease patients during COVID‐19: A UK cohort study

**DOI:** 10.1002/jgh3.12699

**Published:** 2022-01-05

**Authors:** Raphael P Luber, Alexa Duff, Polychronis Pavlidis, Sailish Honap, Susanna Meade, Shuvra Ray, Simon H Anderson, Joel Mawdsley, Mark A Samaan, Peter M Irving

**Affiliations:** ^1^ Department of Gastroenterology Guy's and St Thomas' NHS Foundation Trust London UK; ^2^ School of Immunology and Microbial Sciences King's College London London UK

**Keywords:** anxiety, COVID‐19, depression, inflammatory bowel disease, pandemic, stress

## Abstract

**Background and Aim:**

Patients with chronic diseases are believed to be at increased risk of mental health conditions during the COVID‐19 pandemic. We aimed to assess the incidence of psychological morbidity in inflammatory bowel disease (IBD) patients during the COVID‐19 pandemic, explore for association with risk of severe COVID‐19 and other factors, and establish patients' interest in psychological support.

**Methods:**

A survey including the Patient Health Questionnaire‐9, General Anxiety Disorder‐7, and Perceived Stress Scale tools for depression, anxiety, and stress was administered to IBD patients from a tertiary center in London, United Kingdom, in June 2020.

**Results:**

Two hundred seventy‐four patients responded to the survey (57% response rate), with 271 (99%) completing it. Moderate–severe depression was observed in 61 (22.5%), while 49 (18%) had moderate–severe anxiety; 39 (14%) had both diagnoses. Mean (SD) stress score was 16.2 (7.4). There was no association between degree of severe COVID‐19 risk and psychological morbidity. Flare symptoms and fatigue were associated with worse psychological morbidity, while accessibility of information regarding COVID‐19 risk and reducing that risk was protective for depression (odds ratio [OR] 0.56 [0.33–0.94], *P* = 0.03), anxiety (OR 0.62 [0.4–0.96], *P* = 0.03), and stress (standardized β‐coefficient −0.15 [−0.28 to −0.03], *P* = 0.02). Seventy‐nine (30%) respondents were interested in receiving psychological support during the pandemic, while 200 (76%) expressed interest beyond the pandemic.

**Conclusions:**

Although depression, anxiety, and stress among IBD patients during the pandemic were common, their frequency was similar to pre‐pandemic rates and recent general population levels. Ensuring easy access to personalized risk information with targeted psychological support may mitigate psychological burden as patients reintegrate into society and deal with future COVID‐19 waves.

## Graphical abstract

In a cohort of inflammatory bowel disease (IBD) patients during the COVID‐19 pandemic, at least moderate depression and anxiety were common, but at similar rates to pre‐pandemic IBD data, and recent general population data. Accessibility of information regarding COVID‐19 risk and reducing that risk appeared protective for depression, anxiety, and stress. There was strong interest in receiving psychological support during and beyond the pandemic.

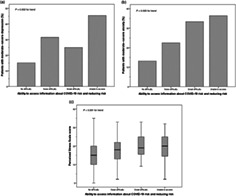

## Introduction

The COVID‐19 pandemic has affected and led to the deaths of millions worldwide. The United Kingdom, particularly impacted, has undergone a rapid and unprecedented change in the functioning of society. As a result, significant negative mental health impacts associated with COVID‐19 have been predicted,^1^ with studies published already supporting these concerns.^2^, ^3^, ^4^, ^5^

The negative impacts of past pandemics on mental health are well recognized, with some studies suggesting lasting effects.^6^, ^7^ It has been hypothesized that those with compromised immune systems or other preexisting medical or psychiatric disorders may be at heightened risk of the psychological effects of the COVID‐19 pandemic.^1^ Supporting this concern, three general population studies during COVID‐19 have found poorer psychological health associated with age, self‐perceived COVID‐19 risk, and a history of undefined medical problems.^2^, ^3^, ^4^ However, psychological morbidity during a pandemic in a population with a chronic disease on immune‐modulating treatments, specifically, is unclear.

Patients with inflammatory bowel disease (IBD), frequently treated with immune‐modulating medication, are both at increased risk of infection^8^ and psychological comorbidity.^9^, ^10^, ^11^, ^12^ In a recent systematic review, pooled rates for anxiety and depression in IBD were 20.5 and 15.2%, respectively,^10^ much higher than general population norms of approximately 5% for both depression and anxiety.^13^, ^14^ The relationship between psychological comorbidity and IBD is complex, with evidence for bidirectional effects between psychological morbidity and IBD activity.^12^ Active IBD is associated with higher rates of depression, anxiety, and psychological distress generally,^10^, ^11^, ^12^, ^15^ and prospective studies have shown a link between depression and subsequent disease flares and lack of response to therapy.^16^, ^17^ Psychological comorbidity is also associated with reduced adherence to treatment recommendations.^18^, ^19^

On 11 March 2020, the World Health Organization declared COVID‐19 a pandemic. On 22 March, the British Society of Gastroenterology (BSG) published its advice for the management of IBD patients during the COVID‐19 pandemic,^20^ with the country entering a nationwide lockdown the following day. Patients were divided into high, moderate, and low risk for COVID‐19 complications based on age, comorbidities, IBD activity, and disease‐related therapies. High risk patients were advised via letter or email to “shield,” recommending them to avoid all face‐to‐face contact and not to leave their homes for any reason except for attending medical care. Patients in the moderate and low risk categories were recommended to undertake “stringent” and standard social distancing respectively. “Stringent” social distancing was not otherwise specifically defined.

It is within this context that we aimed to (i) establish the rates of anxiety, depression, and stress among a cohort of IBD patients during the COVID‐19 pandemic by administering validated psychology questionnaires, (ii) explore whether these psychological conditions were associated with patients' BSG‐determined degree of risk of poor outcome from COVID‐19 as well as other biopsychosocial factors obtained through questionnaire, and (iii) ascertain patient interest in psychological intervention in order to establish the demand on our psychological services.

## Methods

### Study design

A survey (Supplementary section 1) including demographic, IBD‐specific, and psychosocial questions was constructed by a multidisciplinary team and uploaded to the online SurveyMonkey platform (SurveyMonkey, San Mateo, United States). No identifiable data were collected. The questionnaire included the validated Patient Health Questionnaire‐9 (PHQ‐9), General Anxiety Disorder‐7 (GAD‐7), and 10‐point Perceived Stress Scale (PSS) questionnaires. The PHQ‐9 is a 9‐question screening tool and measure of severity for depression based on the preceding 2 weeks. Scores 0–4 associate with “none or minimal” depression, 5–9 with “mild” depression, 10–14 with “moderate” depression, 15–19 with “moderately‐severe” depression, and 20–27 with “severe” depression.^21^ The GAD‐7 is a 7‐question screening tool and measure of severity for anxiety, also based on the preceding 2 weeks. Scores 0–4 associate with “none or minimal” anxiety, 5–9 with “mild” anxiety, 10–14 with “moderate” anxiety, and 15–21 with “severe” anxiety.^22^ The PSS is a 10‐question tool generating a global stress score based on the preceding month, and is one of the most widely used methods for assessing and comparing psychological stress.^23^ Higher scores indicate greater levels of perceived stress, with scores ranging between 0 and 56. It is not validated as a diagnostic instrument, and hence there are no associated cutoffs.

The survey was emailed to IBD patients on the IBD email database at Guy's and St Thomas' Hospitals, London, on 1 June 2020, with a reminder email sent on 8 June 2020. The survey closed for analysis on 15 June 2020.

### Study population

Following the publication of the BSG COVID‐19 risk stratification matrix for IBD patients, we stratified all our patients into low‐, moderate‐, and high‐risk groups (Supplementary section 2). “High risk” patients were subsequently contacted via letter to advise them to “shield,” as per government recommendations. “Moderate” and “low” risk patients were not informed proactively; however, risk categories were available to them on patient advocacy websites and they were informed of their risk status if they made contact with our service. All survey respondents were included in the study.

### Statistical analysis

Descriptive statistics were used to summarize basic demographic, clinical, and psychosocial characteristics and outcomes. Percentages refer to the proportion of patients who completed the corresponding question or psychological tool. Categorical variables were compared using the Pearson Chi‐squared test or Fisher's exact test as appropriate. Multiple continuous variables were compared using the Kruskal–Wallis test for nonparametric data, or the one‐way analysis of variance for normally distributed data. To account for order within independent variables, the Jonckheere–Terpstra test was used for continuous outcomes and the linear‐by‐linear association test for categorical outcomes.

With respect to analyzing factors associated with the psychological outcomes, both depression and anxiety scores were dichotomized to “none or mild” (scores 0–9) and “at least moderate” (scores ≥ 10) anxiety or depression. These cutoffs were considered to be most clinically relevant, and in‐line with previous literature.^9^ The PSS was treated as a continuous variable for the purpose of analysis.

Binary logistic regression was used for univariate analysis of continuous variables in which there was a binary outcome, and for multivariate analysis of continuous and categorical variables with a binary outcome. Linear regression was performed for univariate and multivariate analysis of factors with a continuous outcome (i.e. PSS score). Multivariable analyses were performed using the enter method. Those variables with *P* < 0.10 in univariate analysis, or with a hypothesized biopsychosocial relationship to the outcome, were included in the multivariate models.

A *P* value <0.05 was considered statistically significant. Statistical analyses were performed using SPSS v.23 (IBM corporation, Armonk, NY, USA).

### Ethical considerations

Formal ethics committee approval was not required as the survey was for the purpose of service evaluation and improvement.^24^

## Results

### Patient characteristics

The survey was sent to 81 low risk, 292 moderate risk, and 108 high risk patients with responses from 54 (67%), 152 (52%), and 68 (63%) patients from each group, respectively (total *n* = 274, greater proportion of survey responders among low risk *versus* moderate risk, *P* = 0.02). Two hundred seventy‐one (99%) patients completed the entire survey. Demographics are summarized in Table 1. There were a number of demographic and disease‐based differences between the risk groups. There was a higher proportion of male patients in the high risk compared with moderate and low risk groups (*P* = 0.01 and 0.03 respectively), while median age was higher in both the low‐ and high‐risk groups compared with moderate risk (*P* = 0.001 and <0.001, respectively). High‐risk patients had a longer disease duration relative to moderate (*P* < 0.001) and low risk (*P* = 0.02), with a median (interquartile range) of 20 years (10–29.3). Low risk patients were more likely to be on 5‐ASA therapy, while none were on immunosuppressants, in keeping with the BSG risk stratification. There was a higher rate of past surgery among high‐risk compared with low‐risk groups (*P* = 0.005). The rate of symptom flare since March 2020 did not differ between groups, nor the rate of preexisting medication use for depression or anxiety. Only two patients were prescribed anti‐depressive or anti‐anxiety medications after March 2020, both low risk.

**Table 1:** Patient demographics

Variables	All patients (*n* = 274)	Low risk (*n* = 54)	Moderate risk (*n* = 152)	High risk (*n* = 68)	Between groups comparison *P* value
Male, *n* (%)	123 (44.9)	21 (38.9)	62 (40.8)	40 (58.8)^†^,^‡^	**0.03**
Age, years, median (IQR)	39 (31–51)	44 (36–55.5)^‡^	37 (30–48)	44.5 (35.3–58)^‡^	**<0.001**
Years since diagnosis, median (IQR)	12 (7–20)	14 (5.8–21)	10 (6–18)	20 (10–29.3)^†^ ^,^ ^‡^	**<0.001**
IBD subtype					**0.03**
Crohn's disease, *n* (%)	177 (64.6)	29 (53.7)	99 (65.1)	49 (72.1)	
Ulcerative colitis, *n* (%)	86 (31.4)	21 (38.9)	46 (30.3)	19 (27.9)	
IBD‐unclassified, *n* (%)	9 (3.3)	2 (3.7)	7 (4.6)	0 (0)	
Patient unsure, *n* (%)	2 (0.7)	2 (3.7)^‡^	0 (0)	0 (0)	
Current biologic or tofacitinib use, *n* (%)	157 (57.3)	0 (0)	104 (68)^†^	53 (77.9)^†^	**<0.001**
Immunomodulator use, *n* (%)	118 (43.1)	0 (0)	92 (60.5)^†^ ^,^ ^§^	26 (38)^†^	**<0.001**
Current prednisolone use, *n* (%)	9 (3.3)	0 (0)	3 (2)	6 (8.8)^†^ ^,^ ^‡^	**0.01**
Rectal or oral 5‐ASA use, *n* (%)	71 (25.9)	24 (44.4)^‡^ ^,^ ^§^	33 (21.7)	14 (20.6)	**0.002**
Previous surgery (luminal or perianal), *n* (%)	108 (39.4)	14 (25.9)	59 (38.8)	35 (51.5)^†^	**0.02**
Flare of symptoms since March 2020, *n* (%)	60 (21.9)	12 (22.2)	27 (17.8)	21 (30.9)	0.25
Medication for depression or Anxiety, *n* (%)	33 (12.1)	8 (14.8)	16 (10.5)	9 (13.2)	0.65
Prescribed pre‐March 2020	31 (11.3)	6 (11.1)	16 (10.5)	9 (13.2)	
Prescribed post‐March 2020	2 (0.7)	2 (3.7)	0 (0)	0 (0)	

^†^ Higher compared with low risk, *P* value <0.05.

^‡^ Higher compared with moderate risk, *P* value <0.05.

^§^ Higher compared with high risk, *P* value <0.05.

Boldface has been used to highlight *P* values <0.05.

IBD, inflammatory bowel disease; IQR, interquartile range.

Psychosocial factors and their association with risk groups are summarized in Table 2. There was a statistically significant positive association between perceived financial difficulty and higher risk category. This was accompanied by a trend toward higher loss of employment or furlough (temporary leave of absence) with increased risk group, however this did not achieve statistical significance. As expected, there was a rising proportion of patients “shielding” among increasing risk groups; however, only 45.3% of the high‐risk group followed shielding guidance despite governmental recommendations.

**Table 2:** Association between psychosocial factors and risk groups

Variables	All patients (*n* = 274)	Low risk (*n* = 54)	Moderate risk (*n* = 152)	High risk (*n* = 68)	Between groups comparison *P* value	*P* value for trend
Experiencing financial difficulty during COVID, *n* (%)	38 (14.3)	4 (7.8)	20 (13.4)	14 (21.5)	0.10	**0.03**
Lost job or been furloughed during COVID, *n* (%)	37 (18)	4 (11.1)	23 (17.7)	10 (25.6)	0.26	0.10
Moved away from usual home during COVID, *n* (%)	33 (12.5)	5 (9.8)	22 (14.8)	6 (9.4)	0.45	0.86
Shielded, *n* (%)	59 (22.3)	4 (7.8)	26 (17.4)	29 (45.3)^†^ ^,^ ^‡^	**<0.001**	**<0.001**
Suffered bereavement since COVID crisis, *n* (%)	26 (9.8)	5 (9.8)	9 (6.0)	12 (18.8)^‡^	**0.02**	0.07
Using a psychological intervention, *n* (%)	84 (31.9)	19 (37.3)	44 (29.5)	21 (33.3)	0.57	0.71

^†^ Higher compared with low risk, *P* value <0.05.

^‡^ Higher compared with moderate risk, *P* value <0.05.

Boldface has been used to highlight *P* values <0.05.

### Depression, anxiety, and stress among risk groups

**Figure 1:**
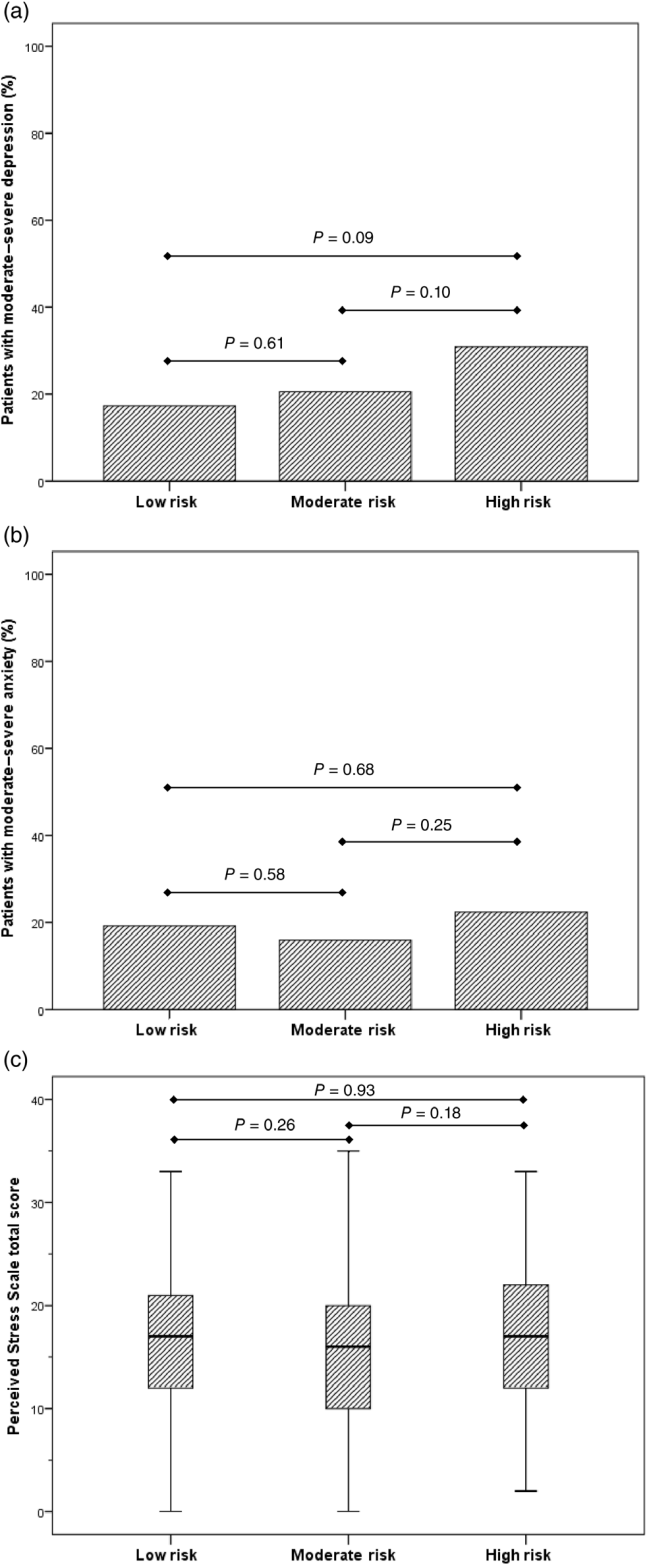
Rates of at least moderate depression (a), anxiety (b), and Perceived Stress Scale scores (c) stratified by COVID‐19 risk group.

Overall, 61 patients (22.5%) had at least moderate depression (PHQ‐9 score ≥ 10), 49 (18%) had at least moderate anxiety (GAD‐7 ≥ 10), and 39 (14%) had both moderate–severe depression and anxiety. Mean (SD) PSS score was 16.2 (7.4). There was no significant difference in scores nor their distribution among psychological severity categories between risk groups (Table 3). Furthermore, there was no significant difference in the rate of at least moderate depression or anxiety across risk groups (Fig. 1).

**Table 3:** Depression, anxiety and stress, stratified by risk category

	All patients (*n* = 271)	Low risk (*n* = 52)	Moderate risk (*n* = 151)	High risk (*n* = 68)	Between groups comparison *P* value	*P* value for trend
PHQ‐9						
Median (IQR)	5 (2–9)	5 (2–8.8)	5 (2–9)	6 (2–11)	0.23	0.4
PHQ‐9 depression category						
None, *n* (%)	118 (43.5)	24 (46.2)	70 (46.4)	24 (35.3)	0.56	0.06
Mild, *n* (%)	92 (33.9)	19 (36.5)	50 (33.1)	23 (33.8)		
Moderate, *n* (%)	36 (13.3)	6 (11.5)	19 (12.6)	11 (16.2)		
Moderately‐severe, *n* (%)	17 (6.3)	2 (3.8)	7 (4.6)	8 (11.8)		
Severe, *n* (%)	8 (3.0)	1 (1.9)	5 (3.3)	2 (2.9)		
GAD‐7						
Median (IQR)	4 (1–7)	5 (1.3–8.5)	3 (1–7)	5 (2–8)	0.30	0.50
GAD‐7 anxiety category						
None, *n* (%)	140 (51.9)	25 (48.1)	85 (56.3)	30 (44.8)	0.80	0.58
Mild, *n* (%)	81 (30)	17 (32.7)	42 (27.8)	22 (32.8)		
Moderate, *n* (%)	25 (9.3)	5 (9.6)	12 (7.9)	8 (11.9)		
Severe, *n* (%)	24 (8.9)	5 (9.6)	12 (7.9)	7 (10.4)		
PSS						
Mean (SD)	16.2 (7.4)	16.9 (7.7)	15.6 (7.3)	17 (7.6)	0.30	0.69

GAD‐7, General Anxiety Disorder‐7; IQR, interquartile range; PHQ‐9, Patient Health Questionnaire‐9; PSS, Perceived Stress Scale.

### Predictors of depression, anxiety, and stress

The clinical and psychosocial predictors of depression, anxiety, and stress are summarized in Table 4. COVID‐19 risk category was not associated with any of the psychological outcomes on regression analyses. Of the factors found to be associated with moderate to severe depression on univariate analysis, only fatigue, patient suspicion of a flare, using a psychological intervention, and difficulty accessing risk information remained statistically significant on multivariate analysis. With respect to predictors of moderate‐to‐severe anxiety, being on medication for anxiety or depression, patient suspicion of a flare, fatigue, and difficulty accessing risk information were statistically significant predictors both on univariate and multivariate analyses. Regarding stress, fatigue showed the strongest association with a higher PSS score, followed by financial difficulties, use of a psychological intervention, and increasing number of people in one's household. Ability to access risk information was protective against stress.

**Table 4:** Factors and their association with psychological outcomes

Factor	Outcome											
	Moderate to severe depression				Moderate to severe anxiety				Perceived Stress Scale score			
	Univariate		Multivariate (*R* ^2^ = 0.26)		Univariate		Multivariate (*R* ^2^ = 0.18)		Univariate		Multivariate (*R* ^2^ = 0.31)	
	OR (95% CI)	*P*	OR (95% CI)	*P*	OR (95% CI)	*P*	OR (95% CI)	*P*	Standardized β‐coefficient (95% CI)	*P*	Standardized β‐coefficient (95% CI)	*P*
Age	0.98 (0.96–1.01)	0.16			0.99 (0.97–1.02)	0.54			−0.09 (−0.21 to 0.03)	0.14		
Years since diagnosis	1.00 (0.98–1.03)	0.88			1.00 (0.97–1.03)	0.93			0.02 (−0.10 to 0.14)	0.74		
Female sex	1.11 (0.63–1.98)	0.72			1.33 (0.71–2.5)	0.38			0.11 (−0.01 to 0.23)	0.08	0.10 (−0.03 to 0.22)	0.13
CD *versus* UC	1.22 (0.65–2.31)	0.53			0.57 (0.27–1.19)	0.13			−0.08 (−0.20 to 0.05)	0.23		
Risk category	1.51 (0.97–2.34)	0.07	0.74 (0.32–1.68)	0.47	1.14 (0.71–1.82)	0.59	0.99 (0.57–1.73)	0.97	0.01 (−0.11 to 0.14)	0.82	−0.08 (−0.21 to 0.05)	0.21
Biologic or tofacitinib	1.9 (1.02–3.41)	**0.04**	2.51 (0.88–7.19)	0.09	1.5 (0.79–2.86)	0.22			0.05 (−0.07 to 0.17)	0.43		
Immunomodulator	0.6 (0.34–1.11)	0.10			0.87 (0.46–1.62)	0.65			−0.08 (−0.20 to 0.04)	0.20		
Prednisolone use	1.76 (0.43–7.25)	0.43			1.3 (0.26–6.46)	0.75			−0.04 (−0.16 to 0.09)	0.57		
Previous surgery	1.08 (0.61–1.94)	0.76			1.08 (0.58–2.03)	0.81			0.003 (−0.12 to 0.12)	0.97		
Fatigue	6.92 (3.32–14.42)	**<0.001**	8.2 (2.83–23.8)	**<0.001**	5.52 (2.55–11.96)	**<0.001**	4.01 (1.75–9.15)	**0.001**	0.36 (0.24–0.47)	**<0.001**	0.26 (0.13–0.38)	**<0.001**
Medication for depression/anxiety	2.57 (1.20–5.54)	**0.02**	0.56 (0.11–2.74)	0.47	3.63 (1.66–7.94)	**0.001**	2.96 (1.18–7.45)	**0.02**	0.18 (0.07–0.30)	**0.003**	0.02 (−0.11 to 0.14)	0.77
Patient suspicion of flare	6.26 (3.38–11.60)	**<0.001**	4.40 (1.86–10.37)	**0.001**	3.98 (2.09–7.57)	**<0.001**	2.86 (1.39–5.85)	**0.004**	0.22 (0.01–0.34)	**<0.001**	0.05 (−0.07 to 0.18)	0.40
Shielded	3.0 (1.59–5.66)	**0.001**	2.00 (0.74–5.37)	0.17	2.01 (1.01–3.99)	**0.04**	1.47 (0.63–3.46)	0.37	0.12 (−0.004 to 0.24)	0.06	0.06 (−0.06 to 0.19)	0.33
Moved away from home	1.12 (0.48–2.66)	0.78			1.53 (0.64–3.63)	0.34			0.004 (−0.12 to 0.13)	0.95		
Financial difficulties	6.98 (2.38–20.5)	**<0.001**	1.26 (0.36–4.47)	0.72	1.45 (0.64–3.3)	0.37			0.30 (0.18–0.41)	**<0.001**	0.23 (0.09–0.36)	**0.001**
Lost job or been furloughed	2.21 (0.99–4.88)	**0.047**	1.93 (0.61–6.07)	0.26	1.22 (0.49–3.06)	0.67			0.11 (−0.03 to 0.25)	0.11	−0.02 (−0.15 to 0.11)	0.78
Number of people in household	0.92 (0.72–1.17)	0.50			0.95 (0.73–1.23)	0.69			0.09 (−0.03 to 0.21)	0.15	0.13 (0.01–0.25)	**0.04**
Suffered bereavement	2.41 (1.03–5.64)	**0.04**	1.15 (0.29–4.60)	0.84	3.29 (1.39–7.8)	**0.005**	1.78 (0.60–5.26)	0.30	0.14 (0.01–0.26)	**0.03**	0.04 (−0.09 to 0.16)	0.57
Using a psychological intervention	1.88 (1.03–3.42)	**0.04**	2.48 (1.00–6.17)	0.05	2.19 (1.15–4.16)	**0.02**	2.04 (0.97–4.28)	0.06	0.28 (0.16–0.39)	**<0.001**	0.22 (0.09–0.34)	**0.001**
Ease of access to COVID‐19 risk information	0.58 (0.41–0.82)	**0.002**	0.56 (0.33–0.94)	**0.03**	0.60 (0.42–0.87)	**0.007**	0.62 (0.40–0.96)	**0.03**	−0.23 (−0.35 to −0.11)	**<0.001**	−0.15 (−0.28 to −0.03)	**0.02**

CD, Crohn's disease; CI, confidence interval; OR, odds ratio, UC, ulcerative colitis. Boldface has been used to highlight *P* values <0.05.

**Figure 2:**
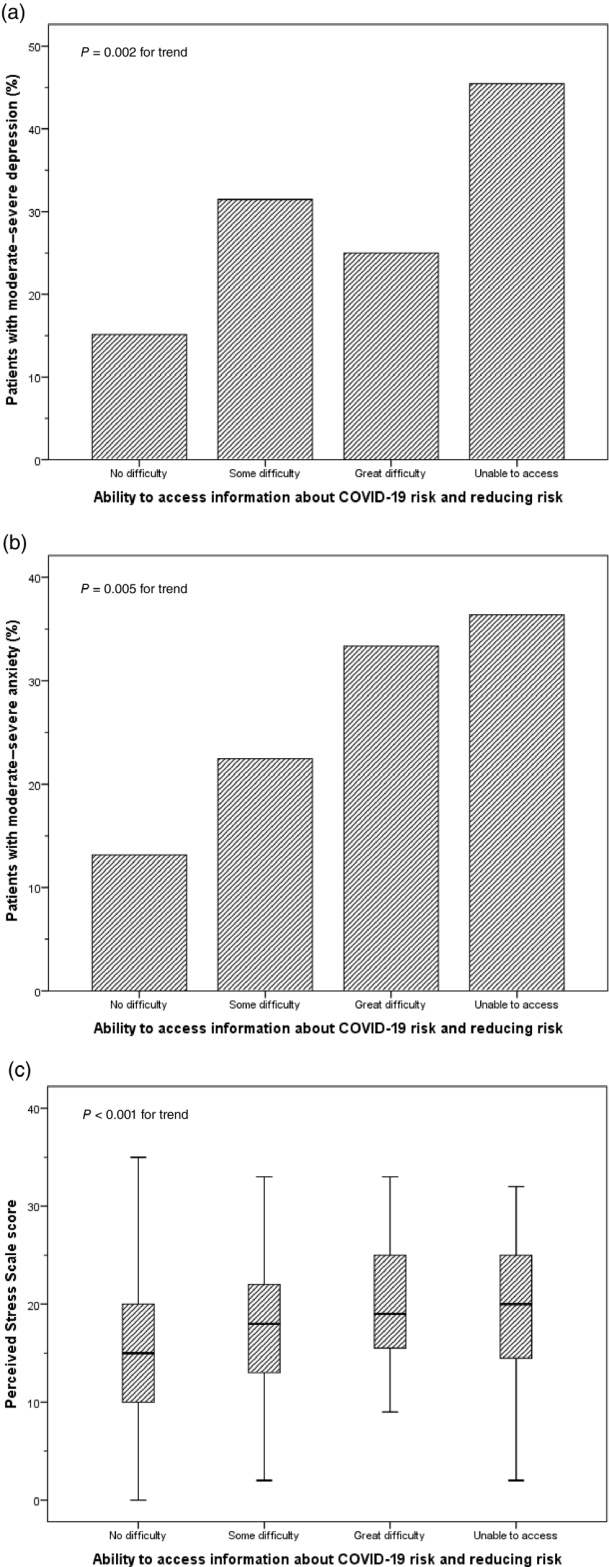
Association between ability to access information regarding COVID‐19 risk and how to reduce that risk, and at least moderate depression (a), anxiety (b), and Perceived Stress Scale score (c).

The self‐assessed degree of difficulty accessing information about personal COVID‐19 risk was also strongly associated with at least moderate depression (*P* = 0.002 for trend), at least moderate anxiety (*P* = 0.005 for trend), and perceived stress score (*P* < 0.001 for trend) (Fig. 2).

### Interest in psychological intervention

There was strong interest in receiving psychological input. Seventy‐nine (30%) participants were interested in therapy during the COVID‐19 pandemic, with 64.6% preferring the interaction to occur via telephone or video call as opposed to face‐to‐face (35.6%). Regarding psychological therapy beyond the pandemic period, there was definite interest from 80 (30.4%) and possible interest from another 120 (45.6%) patients. There was no difference in interest across risk groups for either psychological input during COVID‐19 (*P* = 0.73) or in the future (*P* = 0.96). Most patients open to future therapy expressed interest in individual therapy (*n* = 161, 81%) as opposed to group therapy (*n* = 45, 23%), with a large portion open to online therapy options (*n* = 119, 60%).

## Discussion

Since the beginning of the COVID‐19 pandemic, publications have either predicted or reported significant negative mental health effects related to the pandemic.^1^, ^2^, ^3^, ^5^, ^6^ Some groups have been predicted to be at heightened risk of poor mental health outcomes, including those with preexisting medical or mental health conditions, particularly those with compromised immune systems, and the elderly,^1^ although limited evidence exists thus far to support this concern. Accordingly, this study reports the rates of depression, anxiety, and stress in a population with a chronic condition associated with use of immunosuppression, namely IBD, during the COVID‐19 pandemic. Risk category for severe COVID‐19 was not associated with worse psychological outcomes, although other predictors were identified as was a desire for psychological support.

The overall prevalence of at least moderate depression and anxiety was 22.5 and 18%, respectively. Interestingly, these rates are similar to previously reported rates among international IBD cohorts pre‐COVID‐19.^9^, ^15^, ^25^, ^26^ In a recent, albeit smaller, Portuguese study using different measures, rates of at least moderate depression were similar (20.9%), while anxiety was higher (51.6%).^27^ With regard to perceived stress, the overall mean (SD) PSS score among our cohort was 16.2 (7.4), also similar to pre‐COVID‐19 studies.^11^, ^18^

Despite the ominous mental health predictions, there are a number of potential reasons why levels of depression, anxiety, and stress, did not appear worse than usual. Ordinarily, the availability of and proximity to toilet facilities is a significant source of concern for IBD patients, as is the need to take sick leave from work or adjust working patterns.^28^, ^29^ With nationwide stay‐at‐home orders in place and working from home becoming the norm during COVID‐19, these known stressors may have been mitigated.

While typically IBD patients have higher rates of psychological morbidity than the general population, the rates of depression and anxiety in this cohort appear no worse than recent data from general population studies during the pandemic. In a recent general population meta‐analysis, pooled rates of stress, depression, and anxiety using varying definitions were 29.6, 31.9, and 33.7%, respectively.^5^ However, the included studies are likely to have been subject to participation bias, skewing their data toward higher psychological burden, and were performed early during the pandemic when psychological distress was possibly greater. Nonetheless, while IBD patients may ordinarily feel a sense of isolation in dealing with the effects of their disease,^30^ the society‐wide impact of COVID‐19 may provide IBD patients a new sense of inclusivity in the challenges faced, mitigating some of the psychological impact.

Perhaps surprisingly, there was no association between the use of drugs that impact the immune system and psychological outcomes. These findings are congruent with a recent survey of IBD patients attending our infusion unit for biologic medications in which almost 60% expressed no or minimal concern with respect to their risk of being on medication.^31^ While we acknowledge that this is a self‐selecting group who felt comfortable in attending the unit, another study reported only 42% of IBD patients expressed concern about the impact of their medications on COVID‐19.^32^ The general lack of association between immunosuppression and psychological outcomes may relate to patients feeling protected with social distancing and stay‐at‐home measures.

The only IBD‐related factor associated with all adverse psychological outcomes was patient suspicion of active disease. Active disease is known to be associated with psychological morbidity,^9^, ^10^, ^15^ although studies have generally measured patient‐reported symptoms rather than objective disease markers. It is therefore not possible to distinguish between functional symptoms and true inflammation. Regardless, symptomatic patients in particular should be questioned for concurrent mental illness to facilitate targeted psychological intervention. Furthermore, fatigue was strongly associated with all psychological outcomes, and inquiring about this symptom potentially represents a useful pointer toward psychological morbidity.

Patients' reported ease in being able to access information about their COVID‐19 risk and how to reduce that risk was associated with all psychological outcomes. This suggests that a potentially useful intervention to limit the psychological impact of the pandemic may be ensuring easy access to personalized information. This is supported by recent data suggesting access to an IBD‐specific interaction was associated with alleviating concerns, while obtaining information solely from unofficial sources was not.^32^ Conversely, an earlier global survey showed only 11% of patients found relief from their COVID‐19‐related worries following a medical consultation, with more finding reassurance from patient associations (81%) or relatives (53%).^33^ It is possible, however, that being dissatisfied with the information obtained is not causal of psychological morbidity but rather an effect of it. Comorbid anxiety increases threat bias, and therefore additional reassurance will be needed in cases with comorbid anxiety disorder,^34^ if not other psychological morbidity as well. As the world faces current and future waves of the virus, services will need to provide easy to access, rapid, personalized risk assessments and advice with appropriate targeted psychological support, akin to precision medicine.

A large proportion of patients expressed an interest in engaging in psychological therapies at the time of the survey and in the future. Ideally, our services need to meet this demand. The openness of patients to telephone, video call, or online therapy is reassuring given the constraints of healthcare in the pandemic era. Although patients indicated that they would prefer individual therapy to group therapy, group therapy has been shown to be beneficial in those with IBD,^35^ and is cost‐effective.

Our study has a number of limitations. Firstly, as we have no pre‐COVID‐19 psychological data from this cohort, we cannot comment on the specific effect the pandemic has had on their mental health. Similarly, as there is no matched general population control group, one cannot draw firm conclusions regarding the current state of IBD psychological morbidity relative to the general population. Secondly, selection bias may have influenced outcomes. The responding population had few older patients, which may have reduced the power to detect between risk‐group differences, especially given that age >70 years was a defining feature of high‐risk status. Thirdly, while we informed all high‐risk patients of their risk category, those in the low and moderate risk categories were not directly informed; hence, their psychological state may have been worse than it otherwise would have been had they been informed and potentially reassured about their risk state. Furthermore, several of our patients were erroneously sent letters from separate UK governmental organizations incorrectly attributing them to high‐risk status. This misinformation may have influenced psychological outcomes. Fourthly, portions of the survey conducted are not validated. However, given that most non‐validated questions pertain to demographics, we do not predict the lack of validation would have a significant effect on the results. Finally, given the survey relied on patient responses only, some variables including comorbidities not accounted for in the BSG risk stratification matrix, as well as IBD characteristics, were not collected, and hence their influence on outcomes cannot be determined.

In conclusion, this study suggests that while there are high rates of anxiety, depression, and stress among IBD patients during the COVID‐19 pandemic, rates do not appear to be higher than pre‐pandemic levels and are not associated with COVID‐19 risk category. This may change, however, as social restrictions are eased, patients are reintegrated into society, and vaccination is offered, and as such psychological health requires ongoing monitoring. Ensuring easy access to information regarding COVID‐19 risk and reducing that risk may serve to mitigate some psychological burden; however, this needs to be supported by the availability of psychological support, which over 70% of patients expressed an interest in receiving. This will be particularly relevant in the event of future waves or variants of the virus, and ensuring vaccine uptake.

## Supporting information


**Appendix** **S1.** Supporting information.
