# Supplementary material for: Depression, anxiety, and stress among inflammatory bowel disease patients during COVID‐19: A UK cohort study
Source: JGH Open. 2022 Jan 5;6(1):76–84. doi: 10.1002/jgh3.12699 (PMC8762622; doi:10.1002/jgh3.12699)
Supplement: Supplementary file 1 — Appendix S1. Supporting information. [file JGH3-6-76-s001.docx]

**Supplementary Section:**

**1. Questionnaire:**

*Introduction:*

You have been sent this questionnaire because we wish to find out how our patients are managing

during the current time so that we can better inform the psychological support that we provide.

The following questionnaire asks about your IBD, the emotional impact of the current crisis as well as

specific difficulties that you may have experienced.

Your responses are anonymous so unfortunately we cannot respond to individual questionnaires.

Your participation is greatly appreciated and helps us to improve the service we provide.

*Section 1: Information about you and your inflammatory bowel disease*

| 1. What is your sex? | Male  Female |
| --- | --- |
| 2. What is your age in years? |  |
| 3. What type of inflammatory bowel disease do you have? | Crohn's Disease or Crohn's colitis  Ulcerative Colitis or Ulcerative proctitis  IBD-Unclassified / Indeterminate colitis  Unsure |
| 4. To the nearest year, how many years has it been since you were diagnosed with Inflammatory Bowel  Disease? |  |
| 5. Please select which biologic therapy you are currently on. Select "none" if not on one of these medications. | None  Infliximab (Flixabi/Remsima)  Adalimumab (Humira/Idacio)  Golimumab (Simponi)  Vedolizumab (Entyvio)  Ustekinumab (Stelara)  Tofacitinib (Xeljanz)  Not sure |
| 6. Please select which immunomodulator tablet you are currently on. Select "none" if not on any of these  medications. | None  Azathioprine  Mercaptopurine  Methotrexate  Tioguanine  Not sure |
| 7. If you are currently on steroid tablets, please select the type. Select "none" if not on any steroid tablets. | None  Prednisolone*  Budesonide (Entocort, Cortiment)  Clipper  Not sure |
| *8. What daily dose of prednisolone are you currently on? |  |
| 9. Are you on any other medication specifically for your inflammatory bowel disease currently? | No others listed below  Oral or rectal 5-ASA (eg. Mesalazine, Mezavant, Octasa, Pentasa, Asacol, Salofalk, Sulfasalazine, Colazide)  Rectal steroids (eg. Predfoam, Predsol, Budenofalk, Prednisolone suppositories) |
| 10. Have you previously required surgery (either abdominal or perianal) for your inflammatory bowel disease? | Yes  No |
| 11. Do you think you have had a flare since March 1st 2020? | Yes  No  Maybe |
| 12. Are you currently on medication for depression or anxiety? | Yes*  No |
| *13. When was this prescribed for you? | Before March 2020  Since March 2020 |

*Section 2: PHQ 9 - Depression Questionnaire*

Please read each item and select the response which comes closest to how you have been feeling in

the past 2 weeks. Don’t take too long over your replies; your immediate reaction to each item will

probably be more accurate than a long thought out response.

Over the last 2 weeks, how often have you been bothered by any of the following problems?

| 14. Little interest or pleasure in doing things | Not at all  Several days  More than half the days  Nearly every day |
| --- | --- |
| 15. Feeling down, depressed, or hopeless | Not at all  Several days  More than half the days  Nearly every day |
| 16. Trouble falling or staying asleep, or sleeping too much | Not at all  Several days  More than half the days  Nearly every day |
| 17. Feeling tired or having little energy | Not at all  Several days  More than half the days  Nearly every day |
| 18. Poor appetite or overeating | Not at all  Several days  More than half the days  Nearly every day |
| 19. Feeling bad about yourself, or that you are a failure or have let yourself or your family down | Not at all  Several days  More than half the days  Nearly every day |
| 20. Trouble concentrating on things, such as reading the newspaper or watching television | Not at all  Several days  More than half the days  Nearly every day |
| 21. Moving or speaking so slowly that other people could have noticed? Or the opposite — being so fidgety or restless that you have been moving around a lot more than usual | Not at all  Several days  More than half the days  Nearly every day |
| 22. Thoughts that you would be better off dead or of hurting yourself in some way | Not at all  Several days  More than half the days  Nearly every day |

*Section 3: GAD 7 - Anxiety Questionnaire*

Please read each item and circle the reply which comes closest to how you have been feeling in the

past 2 weeks. Don’t take too long over your replies; your immediate reaction to each item will probably be more accurate than a long thought out response.

Over the last 2 weeks, how often have you been bothered by any of the following problems?

| 23. Feeling nervous, anxious or on edge | Not at all  Several days  More than half the days  Nearly every day |
| --- | --- |
| 24. Not being able to stop or control worrying | Not at all  Several days  More than half the days  Nearly every day |
| 25. Worrying too much about different things | Not at all  Several days  More than half the days  Nearly every day |
| 26. Trouble relaxing | Not at all  Several days  More than half the days  Nearly every day |
| 27. Being so restless that it is hard to sit still | Not at all  Several days  More than half the days  Nearly every day |
| 28. Becoming easily annoyed or irritable | Not at all  Several days  More than half the days  Nearly every day |
| 29. Feeling afraid as if something awful might happen | Not at all  Several days  More than half the days  Nearly every day |

*Section 4: PSS – stress questionnaire*

The following questions ask about your feelings and thoughts during the past month. In each

question, you will be asked how often you felt or thought a certain way.

| 30. In the past month, how often have you been upset because of something that happened unexpectedly? | Never  Almost never  Sometimes  Fairly often  Very often |
| --- | --- |
| 31. In the past month, how often have you felt unable to control the important things in your life? | Never  Almost never  Sometimes  Fairly often  Very often |
| 32. In the past month, how often have you felt nervous or stressed? | Never  Almost never  Sometimes  Fairly often  Very often |
| 33. In the past month, how often have you felt confident about your ability to handle personal problems? | Never  Almost never  Sometimes  Fairly often  Very often |
| 34. In the past month, how often have you felt that things were going your way? | Never  Almost never  Sometimes  Fairly often  Very often |
| 35. In the past month, how often have you found that you could not cope with all the things you had to do? | Never  Almost never  Sometimes  Fairly often  Very often |
| 36. In the past month, how often have you been able to control irritations in your life? | Never  Almost never  Sometimes  Fairly often  Very often |
| 37. In the past month, how often have you felt that you were on top of things? | Never  Almost never  Sometimes  Fairly often  Very often |
| 38. In the past month, how often have you been angry because of things that happened that were outside of  your control? | Never  Almost never  Sometimes  Fairly often  Very often |
| 39. In the past month, how often have you felt that difficulties were piling up so high that you could not  overcome them? | Never  Almost never  Sometimes  Fairly often  Very often |

*Section 5:* Living arrangements, lifestyle and finances

| 40. During the COVID crisis, have you been experiencing financial difficulties? | Yes  No |
| --- | --- |
| 41. **Prior** to the COVID crisis, were you working? | Yes*  No |
| *42. During the COVID crisis, have you lost your job or been furloughed? | No  Yes, because of my inflammatory bowel disease or its treatment  Yes, because of COVID-19  Yes, for other reasons |
| 43. What is the total number of people in your household (including yourself)? |  |
| 44. Have you moved away from your usual home during the COVID crisis? | No  Yes* |
| *45. Why did you move away from your usual home during the COVID crisis? | Due to my inflammatory bowel disease alone  Due to COVID alone  Due to both my inflammatory bowel disease AND COVID  Due to other reasons |
| 46. During the COVID crisis, pre-June 1st 2020, did you leave the house for any reason apart from medical appointments? | Yes*  No |
| *47. Do you leave your home to go to work? | Yes  No |
| 48. Is anyone in your household leaving home to go to work? | Yes  No |
| 49. Have you found that you have been more fatigued than usual since the beginning of the COVID crisis? | Yes  No |
| 50. Have you suffered a death (of any cause) of someone close to you since the beginning of the COVID crisis? | Yes  No |

*Section 6: Accessing information and help*

| 51. Have you been able to access information about your COVID infection risk and reducing your risk during  the COVID crisis? | Yes, without any difficulty  Yes, but with some difficulty  Yes, but with great difficulty  No |
| --- | --- |
| 52. Have you been able to access your inflammatory bowel disease monitoring and treatments during the  COVID crisis? | Yes, without any difficulty  Yes, but with some difficulty  Yes, but with great difficulty  No |

*Section 7: Use of psychological therapies now and in the future*

| 53. Are you using any psychological interventions currently? (eg. apps, lessons from previous psychology  sessions, mindfulness) | Yes  No |
| --- | --- |
| 54. Would you be interested in receiving psychological therapy during the COVID crisis? | Yes*  No |
| *55. How would you prefer to meet a therapist? | Face to face  via online video call  via telephone |
| 56. Would you be interested in psychology input in the future? | No  Yes*  Maybe* |
| *57. What type of psychology input would you be interested in? (select all that apply to you) | Individual therapy  Group therapy  Online therapy |

Note, only responses marked with a “*” were followed by the next question marked with a “*”.

**2. British Society of Gastroenterology COVID-19 risk stratification matrix for risk of serious COVID-19** (adapted from Kennedy NA, Jones G-R, Lamb CA*, et al.* British society of gastroenterology guidance for management of inflammatory bowel disease during the covid-19 pandemic. *Gut* 2020;69:984-90)

| **High risk** | **Moderate risk** | **Low risk** |
| --- | --- | --- |
| 1. IBD patients who either have a comorbidity (respiratory, cardiac, hypertension or diabetes mellitus) and/or are ≥70 years old and are on any ‘moderate risk’ therapy for IBD (per middle column) and/or have moderate to severely active disease | 1. Patients on the following medications:  – Anti-TNF (infliximab, adalimumab, golimumab, certolizumab) monotherapy  – Biologic plus immunomodulator (with combined use >6 weeks) in stable patients  – Ustekinumab  – Vedolizumab  – Thiopurines (azathioprine, mercaptopurine, tioguanine)  – Methotrexate  – Calcineurin inhibitors (tacrolimus or ciclosporin)  – Janus kinase (JAK) inhibitors (tofacitinib)  – Immunosuppressive trial medication  – Mycophenolate mofetil  – Thalidomide  – Prednisolone <20mg or equivalent per day | Patients on the following medications:  - 5-ASA  - Rectal therapies  - Orally administered topically acting steroids (budesonide or beclometasone)  - Therapies for bile acid diarrhoea (colestyramine, colesevelam, colestipol)  - Anti-diarrhoeals (eg, loperamide)  - Antibiotics for bacterial overgrowth or perianal disease |
| 2. IBD patients of any age regardless of comorbidity and who meet one or more of the following criteria:  – Intravenous or oral steroids ≥20mg prednisolone or equivalent per day (only while on this dose)  – Commencement of biologic plus immunomodulator or systemic steroids within previous 6 weeks  – Moderate to severely active disease, as judged by their clinical team, not controlled by 'moderate risk’ treatments  – Short gut syndrome requiring nutritional support  – Requirement for parenteral nutrition | 2. Patients with moderate to severely active disease who are not on any of the medications in this column |  |
